# Rachel Challice's Vow

**Published:** 1888-01-14

**Authors:** Lena Neville

**Affiliations:** Author of "A Future on Trust," "Juno," &c.


### Rachel Challice's Vow.

By Lena Neville, Author of "A Future on Trust," "Juno," &c.

### Chapter II.—Bereavement.

"She gazed upon a world she scarcely knew,
Her spirit seemed as seated on a throne,
Apart from the surrounding world, and strong
In its own strength—most strange in one so young."
—*Byron.*

Presently the murmur of voices, the sound of light laughter outside, came echoing on the frosty night air.

It was Esther Challice coming home from her party.

She was not alone. Two young men accompanied her; and walking between them, she flirted and coquetted and thoroughly enjoyed herself; her merry voice and joyous laugh ringing out clearly and unconsciously as she came near the house of death.

"Here we are!" she exclaimed merrily, as the trio reached the cottage door, and her two companions prepared to take leave.

"Good-night, Harry; good-night, David. Thanks so much for seeing me home—I'm always so timid walking by myself at night. I'm sorry I can't ask you in, but it's so late, and Granny will be gone to sleep."

She little realized the real import of her words, or dreamed how true her statement was. Mrs. Challice did sleep—the sound, unwakening sleep of death.

Esther bade good-bye to her cavaliers out there beneath the blue canopy of heaven, with the light of the throbbing stars and the calm moon shining on her pretty soulless face; then she pushed open the cottage door, and walked into the kitchen.

All was gloomy. The fire had gone out, and no candle had been lighted; only the faint moonbeams here and there struggling through the small diamond-paned window lit up the desolate kitchen, making the rest of the room look darker by contrast. Esther hated darkness and solitude.

"Rachel, Rachel!" she called fretfully. "Where are you?"

No response came in answer to her call.

"They've gone to sleep and forgotten me," pouted the spoiled girl. "How stupid of Rachel to be sure; just like her to think of no one but herself! She'll have to wake up, though, and get me a light. I'm not going to grope my way about in this horrid dark without a candle."

Then she caught sight of a line of light beneath the door of the bed-room. She turned the handle and went in.

Rachel still crouched, pale and motionless, by the bedside. She had scarcely moved since the last breath had escaped her grandmother's lips. She was, for the moment, utterly stunned and powerless.

The sound of her sister's voice roused her. The self-restraint and self-forgetfulness she daily practised came instinctively to her aid, and lent her fresh power and nerve. She rose from her knees, and took her sister's hands.

"Poor Granny!" she said softly. "She died half-an-hour ago."

Esther broke into a storm of noisy sobs and tears.

"Granny dead, and you can speak of it like that! Oh, why didn't you send for me to see her? Oh, Rachel, how cruel of you to let her die without coming for me!"

"You were better away, dear," replied Rachel, still more gently, never heeding the injustice of Esther's reproaches; while she drew the girl towards her with a love and tenderness she seldom, if ever before, had allowed herself to display.

Esther responded to the caress with energy, and laid her pretty golden head on her sister's shoulder, and cried on till the first burst of passionate grief was over.

"Now come to bed, dear," said Rachel. "Stay, you would like to kiss her first, wouldn't you?"

She put out her hand to draw away the sheet that covered Mrs. Challice's face, but Esther shrank back, shuddering.

Hers was a fair-weather sort of love, not the kind warranted to stand rough usage or sharp shocks. The life suitable to her shallow nature would have been that of the Lotos-eaters, in that charmed country "where it was always afternoon," where life could be dreamed placidly and happily away with no unpleasant realities to mar its enjoyment.

"Oh, Rachel! cried the spoilt child piteously, "I couldn't. Poor darling Granny! How dreadful she does look. Oh, come away, Rachel! Her face will haunt me all night, and I sha'n't be able to sleep a wink if I look at it longer. I may come into your bed to-night, mayn't I, Rachel? I should be so dreadfully frightened to wake up and feel no one near. I should fancy all sorts of horrible things were coming to me, and poor Granny's face above all."

"Of course, dear," replied her sister.

But Rachel could not enter into Esther's feelings.

If any passionately-loved being of hers had died she would have liked to have watched jealously by the corpse—alone, like Rispah of old, to have guarded the last remnants of humanity till they were torn from her sight, and then the grave of her loved one would have become her altar and her paradise.

She could not comprehend the fearing in death anyone who had been loved in life; such an idea seemed terrible and unnatural to her mind.

She herself had felt the shock of her grandmother's death, but in a different way—merely as the awful, mysterious severance of body and soul must always affect a person not by constant contact inured to the sight. But she had expected Esther's grief to be uncontrollable and intense, as she had believed the girl's love for her grandmother to be excessive. She never looked on anything but what there was of good in her sister's composition; and it puzzled, and in a measure distressed her, when she got up next morning, after a wakeful night, to find Esther sleeping as calmly as though nothing terrible or unusual had occurred.

Such indifference was beyond her comprehension. She could have understood outward coldness in herself. But Esther she fondly believed to be made of far finer, rarer, and more delicate clay; and it perplexed her to find such callousness. She stole softly downstairs, and began to set forward with her household work; for she knew that as soon as possible she must give due notice to the proper authorities of old Mrs. Challice's death, and make arrangements for the funeral.

The pale gleam of the morning was showing over the dusk of the moor as Rachel opened the cottage door.

Hebog and the ranges of mountains about and beyond Ryhd-ddu loomed purple, sullen, and dark against the faint primrose-coloured sky.

Suddenly a shaft of light sprung up in the eastern heavens, a ball of fire shone between the heavy lines of grey cloud that barred the pallid sky, and the golden haze of the rising sun flooded the moor with waves of magical lustre.

Hebog, Arran, Siabod, and Snowdon cast off their night veils of mist and put on their sunny golden crowns to welcome the rising of the new day. Colwyn sang its noisy welcome as it dashed over its rocky bed. Dinas and Gwynant lakes, and even dark melancholy Llyn-Magi, with its boggy shores and sheer descending precipitous mountain girders, rippled and smiled in the bright glow of the frost-touched October morning. The salmon leaped up, sending broad, wide-spreading rings ruffling the glittering surface of the dancing waters; and the sheep-bells tinkled musically as the flocks roamed over their mountain pastures.

A young man came along the road whistling, easting an eager glance at the cottage windows as he passed by. As he did so he caught sight of Rachel.

"You are early this morning," he called out, cheerily, his gaze travelling past her as she spoke, as though searching for something behind.

"Yes," she replied curtly; then added more gently—

"I've a lot to do this morning. Granny died last night."

David Stephens paused as he heard Rachel's news, and stopped his whistling.

"So the poor old woman's gone at last." Then, as Rachel keenly noticed, his glance roamed still more uneasily and earnestly about, while he added hurriedly—

"How does Esther take it?"

A pang shot through the elder girl's heart.

David never thought of *her*.

Yet why should he? All Beth Gelert and Ryhd-ddu knew that it was Esther, not Rachel, who was her grandmother's pet and darling, the very apple of her eye. It was quite natural that young Stephens should think of and inquire first after the child most likely to miss her grandparent.

And there was yet another reason. David Stephens was very much in love with pretty, merry little Esther Challice; and it was only his father's strong disapproval of the match that held him back from openly declaring himself her lover. Old Mr. Stephens would not hear of such a connection, and David was too dutiful a son to set himself in flat contradiction to his father's wishes.

So he consoled himself as best he might with meeting Esther on every possible occasion; and trusting that in the future things might shape themselves more kindly, and his course of true love run smoother than it chose to do at present.

"Esther bears up wonderfully," replied Rachel, bravely making the best of her sister's apparent lack of regret for her loss—a state of mind which, while Rachel was devoutly grateful for it, somehow distressed her, as tending to lower her opinion of her idol.

"Poor child!" she continued; "it was an awful shock for her learning it sudden like when she came home from the Evans's last evening. But she bore it better than I expected."

"Why did you let her find it out like that?" rejoined David reproachfully. "Couldn't you have broken it to her little by little?"

"No," replied Rachel wearily. "I couldn't help it; she came in without my hearing her."

He made no reply; and it was Rachel who presently broke the silence.

"I can't stay longer now, David; I've a lot to do, and not too much time before me."

"And I can't stay either," he replied. "Goodbye, Rachel. Say all that is kind from me to Esther. I'll try and come up this evening and see her, if she's fit to talk to me. Tell her so, and that I couldn't wait for it this morning."

He strode on, and Rachel slowly and heavily went back to her work in the kitchen, her heart sadder than it had been before. A weight she had carried secretly for the past year, above and beyond the petty trials and misunderstandings she daily endured from her own family, seemed suddenly to press with redoubled force on every nerve and fibre of her heart, and to become almost unendurable in its oppression.

A truth that she had, against her better judgment, striven to regard as doubtful, showed itself instantaneously clear and unvarnished before her unwilling gaze.

She loved David Stephens; and she knew, what she had always shrunk from realizing before, that he cared no more for her than the field labourer cares for the dewy gossamer web of the spider, that he ruthlessly brushes away and destroys, as he goes to his work in the morning.

This great hopeless love, with its daily burthen of bitterness and despair, was the grand secret in Rachel Challice's dreary desolate existence.

---

### CHAPTER III.—IT CANNOT BE.

"Oh, madness of discourse,
That cause sets up with, and against itself."
—*Shakespeare.*

The largest building in the little village of Aberclwyn was the mill, and the principal inhabitant of that small colony—in his own estimation—was Mr. Elias Stephens, miller.

To be sure, there was the vicar; and a very sore point it was with the miller that the common people and society in general gave precedence in rank to the Rev. John Jones rather than to Mr. ——, or, as he loved to designate himself, Elias Stephens, Esq. "I don't see it," the old man would argue, doggedly. "Here am I, a landed proprietor, owner of Aberclwyn Mill and twenty cottages besides, to say nothing of three or four fields; and people go and put a vicar before me! A man who hasn't a penny of his own to bless himself with, and lives in a house lent him while he lives! And yet, because he *is* a vicar he thinks himself worth the whole population put together;"

To hear Mr.—we beg his pardon—Elias Stephens, Esq., talk, one would have set him down as an atheist, a socialist, or a rabid Dissenter.

But no. Elias was a firm pillar of the Established Church, a staunch attendant at every service held in Aberclwyn Church, and one who readily undertook the thankless office of churchwarden. The only things he really objected to were burial and graveyard fees; and this hobby he rode so mercilessly that had the Rev. John Jones consented to remit his dues Elias would have forgiven him for being a vicar, and gracefully yielded him the precedence in all matters social or ecclesiastical.

A quiver full of children is, we are informed, a fact calculated to constitute a happy man; but to consummate the joy it is necessary to be certain that your olive branches have a sufficiency of food and clothing, and these were details not quite assured to the young but numerous family of Jones.

So the vicar with his small stipend did not quite see his way to obliging his churchwarden in the matter; and continued to exact his graveyard fees in a way that drove Elias to the very verge of madness, till one day the churchwarden found a vent to his resentment.

When Mrs. Stephens, a weak minded, worthy woman, died she was indeed buried in St. Elwyn's churchyard. But month after month passed, and no funeral urn or granite obelisk marked the site where her ashes lay.

The ground "settled." Time wore on; and one day it was rumoured that the order for a tombstone to her memory had been given to Hughes, the sculptor at Portmadoc.

The rumour became a certainty. Several people saw the monument in the stone-cutter's yard, and a very handsome one they pronounced it,

The stone was finished, dispatched, and duly delivered at the mill.

Mr. Stephens was satisfied. He went to bed happy that night. He had done honour to his wife's memory by ordering and paying for the handsomest monument he could devise, and yet had escaped violating his convictions in refusing to erect the said stone in the churchyard, thus eluding the lawful, but to him intolerable fees.

Instead, it was fixed by Hughes's men in the mill garden, where anyone who chose could read that it was placed there in memory of Eliza Anne, the dearly beloved wife of Elias Stephens, who departed this life on August 30th, 1868, to the inexpressible grief of her sorrowing surviving husband and son.

The Rev. John Jones tried to prove this singular freak on the part of his contumacious churchwarden illegal. But his arguments would not hold water. There is no law to prevent a man erecting a memorial-stone to his wife in his back garden, if he chooses to do so.

Therefore, Elias rested peaceful and happy in the serene consciousness of having done his duty towards the memory of his lost Eliza Anne; and yet of having sacrificed none of his serious convictions by blindly following use and custom. A man holding his head so high, and imbued with so obstinate a nature, was not likely to consent to his only son and heir allying himself with a girl holding so dubious a position as Esther Challice.

He had no personal objection to urge against Esther. She was pretty and winning, and her lack of fortune would have been no insuperable objection in Elias's eyes; but old Stephens held stern notions regarding respectability of birth and antecedents. The visitation of the sins of the fathers on the children, to his thinking, was a good and righteous doctrine, and one he felt it his duty, as a landed proprietor and churchwarden, strictly to uphold. Therefore, to allow David to marry a girl with no legitimate standing would have been a crime of the first magnitude in his eyes, and one not to be tolerated or thought of for an instant. It seemed sheer contrariety on the lad's part to have conceived such an idea of matrimony, and looked in the direction of Esther Challice for a wife, when there was Susan Price—the daughter of old Price of Manorgwyn, whose farm had been in the possession of the family for generations—ready to be had for the asking.

Yes, and Miss Richards too, sister-in-law of the Vicar himself, and whose father was a vicar too—she had looked with kindly eyes on young David, and by coy and blushing, though unmistakable advances, had given him to understand that his suit, if pressed, would not be unwelcome.

David, however, to his father's chagrin, would not so much as glance at the charms of the well-dowered, unimpeachably-descended Miss Price; or on the modestly-willing, clerically-connected Miss Richards.

Esther Challice was the wife he desired, and Esther Challice he would marry; or else, if his Beatrice was denied him, for her sake he would elect to remain a Benedick.

*(To be continued.)*

---

RECENT experiments with the electric light—which of all artificial means of illumination most nearly resembles sunlight—have shown how important a part light plays in promoting growth. At the close of a public lecture it was found that some buds which had remained on the lecture table had actually blown under the influence of the electric light, while others, which had been kept in the dark, had made no progress whatever.—*Malcolm Morris, F.R.C.S.E.*

